# Long-term Prophylaxis with Androgens in the management of Hereditary Angioedema (HAE) in emerging countries

**DOI:** 10.1186/s13023-022-02536-x

**Published:** 2022-11-02

**Authors:** Yinshi Guo, Huanping Zhang, He Lai, Huiying Wang, Herberto J. Chong-Neto, Solange O. R. Valle, Rongfei Zhu

**Affiliations:** 1 Department of Allergy and Immunology, Renji Hospital, Shanghai Jiao Tong University School of Medicine, Shanghai, China; 2 Department of Allergy Medicine, Shanxi Bethune Hospital, Third Hospital of Shanxi Medical University, Shanxi Academy of Medical Sciences, Tongji Shanxi Hospital, Taiyuan, China; 3 Department of Allergy, The Second Affiliated Hospital, Guangzhou Medical University, Guangzhou, China; 4 Department of Allergy, The Second Affiliated Hospital,, Zhejiang University School of Medicine, Hangzhou, China; 5 Department of Pediatrics, Hospital de Clínicas, Federal University of Paraná, Curitiba, Brazil; 6 Department of Clinical Medicine, Hospital Universitário Clementino Fraga Filho, Federal University of Rio de Janeiro, Rio de Janeiro, Brazil; 7 Department of Allergy, Tongji Hospital, Tongji Medical College, Huazhong University of Science and Technology, 430030 Wuhan, China

**Keywords:** Hereditary angioedema (HAE), Androgen, Long-term prophylaxis, Efficacy, Safety

## Abstract

Hereditary angioedema (HAE) is a rare autosomal dominant genetic disease characterized by repetitive subcutaneous or submucosal angioedema, activation of the kinin system, and increased vascular permeability. C1-inhibitor (C1-INH) deficiency, the main mechanism of HAE pathogenesis, occurs when abnormal activation of plasma kallikrein, bradykinin, and factor XII, or mutation of genes such as *SERPING1* cause quantitative or functional C1-INH defects. Although androgens are not approved for HAE treatment in many countries, they are widely used in China and Brazil to reduce the frequency and severity of HAE attacks. The long-term adverse effects of androgen treatment are concerning for both physicians and patients. Virilization, weight gain, acne, hirsutism, liver damage, headache, myalgia, hematuria, menstrual disorders, diminished libido, arterial hypertension, dyslipidemia, and anxiety/depression are commonly observed during long-term treatment with androgens. These adverse effects can affect the quality of life of HAE patients and often lead to treatment interruption, especially in women and children. In-depth studies of the pathogenesis of HAE have led to the approval of alternative treatment strategies, including plasma-derived C1 inhibitor, recombinant human C1 inhibitor, plasma Kallikrein inhibitor (ecallantide; lanadelumab), and bradykinin B2 receptor antagonist (icatibant), some of which have achieved satisfactory results with mostly non-serious side effects. Therefore, a new standard of medical care may expand possibilities for the management of HAE in emerging countries.

## Background

Hereditary angioedema (HAE) is a rare autosomal dominant genetic disease with reported incidence ranging from 1:10,000 to 1:150,000 [1–3]. Potentially fatal and often misdiagnosed, HAE is mainly characterized by recurrent and unpredictable attacks of subcutaneous edema, commonly involving skin and the gastrointestinal and respiratory systems [4]. In particular, skin and visceral systems are affected by the typically massive local edema accompanying HAE. Abdominal attacks can take the form of significant and invalidating pain, whereas the involvement of upper airways can result in laryngeal swelling, obstruction, and severe life-threatening symptoms, including risk of asphyxiation [5]. Due to the cumulative burden of suffering endured by patients with HAE, it can clearly be considered a catastrophic disease.

The main pathological feature of HAE is a deficiency of functional C1 esterase inhibitor (C1-INH) [6]. Currently, HAE is divided into three subtypes: C1-INH consumption (type I), C1-INH inactivation (type II), and HAE with normal C1-inhibitor (HAE nC1-INH). For the first two types, mutations in the gene encoding C1-INH (*SERPING1*) can lead to its quantitative deficiency (type 1 HAE), causing low C1-INH antigen and functional levels, or elicit normal or elevated C1-INH antigen levels, but low C1-INH functionality (type 2 HAE) [7, 8]. Type 1 HAE, the most common form, occurs in approximately 85% of patients and leads to a 5–40% reduction of normal C1-INH functional activity. Type 2 HAE occurs in about 15% of patients [7]. HAE with normal C1-INH is associated with mutations in other genes. To date, six different gene mutations have been reported in HAE nC1-INH: Factor XII (*F12*), plasminogen (*PLG*), angiopoietin 1 (*ANGPT1*), kininogen 1 (*KNG1*), myoferlin (*MYOF*), and heparan sulfate-glucosamine 3-O-sulfotransferase 6 (*HS3ST6*) [3]. However, the prevalence of HAE globally showed no ethnic- or sex-based differences in some countries, and the correlation between different types of mutations and clinical phenotype is controversial. This points to the importance of the involvement of epigenetic changes and other environmental factors in the pathogenesis of HAE.

The ultimate goals of HAE treatment are to achieve total control of the disease and improve patient quality of life (QoL). Androgens have been widely used as long-term prophylaxis treatment for HAE patients in many countries, such as Brazil and China [9, 10]. Indeed, androgens are the most widely used long-term prophylaxis treatment in Brazil, where major adverse effects associated with continued administration of high-dosage androgen treatment are hepatotoxicity and virilization [9]. Similar adverse effects of danazol, including liver dysfunction and androgen-related manifestations, were reported in China [10]. In this paper, the adverse effect of androgens were illustrated in order to guide clinical management of HAE in emerging countries, in where androgens remain the standard treatment for HAE.

## Androgen treatment in HAE

There are three approaches to treating HAE: on-demand, short-term, or long-term prophylaxis [11]. In recent years, the attenuated androgens has been one of the most commonly used long-term prophylactic treatment in some certain countries[12].

Despite having been shown to increase C1-INH mRNA expression, upregulate C1-INH protein levels and promote accelerated degradation of bradykinin to alleviate the symptoms of angioedema, the exact mechanism of androgens requires further study [13, 14]. In the last decade, androgens are considered to be effective and commonly used for HAE and other diseases, such as aplastic anemia, testicular insufficiency, and anorchidism [15–17].

Previous studies demonstrated that danazol can reduce the frequency and severity of HAE attacks [2, 18, 19]. In 1976, a double-blind randomized study including nine patients with HAE found that of the 47 courses of placebo control treatment, attacks still occurred in 44 courses; whereas, only one attack occurred during 46 danazol treatment courses [19]. More recently, a larger double-blind randomized crossover study confirmed these results, showing that 111 of 118 patients responded to danazol; furthermore, 54 (45.8%) achieved symptom-free status or experienced one attack at most per year. In the remaining patients, symptoms exhibited a mild course [20], demonstrating that danazol is effective for the prevention of HAE attacks.

The shortcomings of androgen use in HAE were also reported in several population-based studies. In 2016, a retrospective cross-sectional study of 650 subjects found that 186 subjects discontinued androgen treatment. One of the predominant reasons was the lack of efficacy [21]. Considering these findings, it could be speculated that some adverse effects of androgens were related to variations in treatment methods and/or the severity of disease in HAE patients:

(1) **Dosage of treatment.** Hospital admissions were significantly higher for patients administered 100 mg/day compared with 200 mg/day [21]. In an analysis of androgen dosage that included 16 HAE patients without prior treatment, 15 patients were asymptomatic on administration of 5 mg oxymetholone daily. Among these patients, seven experienced attacks when their schedule was tapered to 5 mg every other day. This result indicates that a higher dosage of androgens was needed to control symptoms in some cases.

(2) **A history of taking androgens.** Androgens are reportedly less effective in patients who had taken these therapies before compared with patients who were androgen-naïve [21].

(3) **Patient compliance.** There were significantly fewer attacks in patients who took the drug continuously compared with patients who used it intermittently [21].

## Adverse effects of androgen treatment

Despite the effectiveness of androgens, adverse effects frequently occur during treatment, including excessive hair growth, weight gain, menstrual disorders, and other conditions [22, 23]. Attenuated androgen (danazol) remains the primarily administered drug in certain areas, especially places where other drugs are not available, such as China [9]. A Chinese survey of 107 patients with HAE reported that 87% used androgens for long-term prophylaxis, of which 50 (74.6%) experienced significant adverse effects. In addition, 75% of female patients experienced menstrual disorder symptoms, 61.8% of patients experienced weight gain, and 41.2% of patients experienced joint pain [24]. In a study of the effects of androgens in 118 patients, adverse effects occurred in 93 (79%) of patients and led to discontinuation of danazol therapy in 30 patients (25%) [20]. The dosage was closely related with the adverse effects mentioned above [25]. In a study including 31 female patients treatment with danazol, six patients stopped treatment due to intolerance of the drug even when the average dosage was 131.7 mg/day [26]. In addition, a retrospective cross-sectional study reported that 73.3% of patients who stopped androgens felt that the adverse effects outweighed the benefits, while 80.2% of patients who remained on androgens felt that the adverse effects outweighed the benefits [21]. However, as the clinical consequences of symptomatic treatment-related adverse effects are often ignored, it is necessary to have a clear understanding of the possible adverse effects of androgens used in HAE patients. It should be noted that, despite some of the clinical data in this review was not extracted from emerging countries, the data could also reflect the current status in China and Brazil, considering the adverse effects of androgen should be consistent in the overall counties and races.

### Virilization

Virilization is the most common adverse effect during androgen treatment, making long-term use of these drugs difficult [23]. Specific changes of virilization include menstrual disorders, decreased libido, and hirsutism. Menstrual disorders usually occur after treatment lasting 3–5 months, with changes directly related to increased levels of androgen in the body [27]. These types of complications are also common in other diseases treated with androgens and especially serious in adolescents because of the influence of hormones on growth [28]. As previously reported, the occurrence of virilization-related adverse effects is as high as 20%, among which hirsutism, weight gain, and menstrual disorders are the most common. Although there is no correlation between the dosage of androgen and symptoms, there may be a positive correlation between the duration of use and symptoms, which sometimes disappear after stopping the drug [26, 27]. A prospective study of 27 patients with HAE (15 females) found that 60% had menstrual disorders after taking a maintenance dose of stanozolol, and approximately 30% continued to have menstrual abnormalities after stopping treatment [29]. In patients with hormone therapy-induced masculinization, androgen therapy is usually discriminately stopped after weighing the pros and cons of symptoms.

### Weight gain

Weight gain is one of the most common adverse effects of androgen therapy. Use of androgens induces poor anabolism by promoting the assembly of complex metabolites, which complicates long-term preventive treatment [30]. The resulting weight gain can only be controlled with a strictly disciplined diet and appropriate exercise. The average weight gain reported in most studies was 2–5 kg. There were also individual gains of more than 10 kg during a prospective follow-up of 69 patients with HAE who received danazol (50–600 mg/day) for 1 to 6 years [31]. Thus, there is a positive correlation between weight gain and higher drug dosage.

### Acne and hirsutism

Androgens affect many organ systems, including the skin. In female patients, the effects of androgens on the sebaceous unit of hair follicles can cause acne, hirsutism, and androgenic alopecia, also known as female pattern hair loss [32]. Clinically, elevated androgen levels are considered a general risk factor for seborrheic dermatitis, and the occurrence of acne is related to androgen or androgen/estrogen levels. Changes in appearance caused by dermatitis or acne may affect a patient’s daily life and cause psychological problems [33, 34], with dose-related severity [1].

### Liver damage

Alkylated androgens exhibit delayed liver metabolism and alkylation, which apparently increase liver toxicity. Reported complications include cholestatic jaundice and elevated liver enzymes [20, 31, 35]. In addition, cases of pancreatitis have been reported [36]. Long-term treatment with androgen derivatives can lead to the development of hepatocellular adenomas and cancer [20, 37]. Bork et al. reported that several patients with HAE developed hepatocellular adenomas after receiving danazol for more than 10 years. In two patients, adenomas disappeared or receded after stopping danazol [37]. A retrospective analysis of 13 patients with HAE found that long-term treatment (15–47 months) with low-dose danazol or stanozolol did not cause significant liver damage detectable by laboratory tests or liver biopsy [38], suggesting a correlation between the dose and occurrence of liver damage. According to clinical studies, long-term prophylactic use of exogenous androgens promotes the formation of hemangiomas, hepatocellular adenomas, and carcinomas, and may cause liver damage or fibrosis [39, 40].

### Headache and myalgia

Headache and myalgia are commonly reported adverse effects of androgen treatment. A retrospective analysis of data from 118 patients with HAE who received danazol for as little as 2 months to 30 years reported that 93 patients (79%) exhibited adverse reactions, 30 (25%) of whom discontinued treatment because of intolerance. Of the 93 patients exhibiting adverse reactions, 16 and 15 experienced headache or myalgia, respectively, but almost all chose to endure the symptoms and continue therapy [1, 20]. Overall, headache and myalgia frequently occurred during androgen treatment of HAE and may affect the QoL of some patients. Androgen therapy can also cause mood changes, including depression and anxiety, that are not entirely dependent on physical changes or the disease itself; rather, adverse effects like headache and myalgia, and an overall poor QoL, are thought to collectively contribute to depression and fatigue [41].

### Hematuria

Hemorrhagic cystitis is reportedly correlated with use of androgens [42]. A 9-year follow-up study of 69 patients with HAE treated with danazol revealed that 13 (19%) developed hematuria and 10 (14%) developed hemorrhagic cystitis. An analysis of the 10 patients with hemorrhagic cystitis showed non-specific patterns of erythema, submucosal telangiectasia, and neovascularization. Bladder biopsy revealed large numbers of dilated submucosal blood vessels with bleeding, mucosal ulcers, and occasional inflammatory cells. Other prospective studies and retrospective case series reported similar rates of hematuria [30, 43]. However, neither the dose nor duration of danazol were related to the severity of hematuria on cystoscopy or pathology, and 90% of patients experienced symptom alleviation after stopping treatment [42].

### Lipoprotein changes

Several studies showed that androgen therapy is related to changes in the lipid atherogenic index, including decreased high-density lipoprotein cholesterol (HDL-C) levels and increased low-density lipoprotein cholesterol (LDL-C) levels; However, there is no consensus on changes in triglyceride levels [44]. A crossover study evaluating the safety of danazol (200 mg/day) in 15 healthy male volunteers over 4 weeks showed that danazol produced a 23% reduction in HDL-C from baseline and 21% reduction in apolipoprotein AI, with no significant differences in other factors [44]. Széplaki et al. later conducted further studies on the effect of androgen (danazol) treatment on the blood lipid profile of 64 patients with HAE. They found that serum concentrations of HDL-C and apolipoprotein AI were significantly reduced in patients receiving danazol, while those of LDL-C and apolipoprotein B100 were increased [45]. However, there is no published evidence indicating that danazol treatment increases the risk of cardiovascular events or atherosclerotic complications. Long-term studies of large populations are required to test these potential effects of danazol treatment.

### Hypertension

In recent years, large doses of androgens were found to be associated with hypertension [46–48]. In 1980, a patient with HAE exhibiting hypertension after danazol treatment was administered small doses of diuretics to normalize their blood pressure level [49]. In 1987, a case report surfaced of three young female patients with HAE who had benign intracranial hypertension when danazol was used, along with simultaneous headache and weight gain [50]. An early study described severe hypertension as an additional adverse effect of danazol. Salt and water retention are believed to be the main reasons for this effect, possibly due to the weak mineralocorticoid properties of danazol. However, the mechanism by which androgen anabolic steroids cause or aggravate high blood pressure is multi-factorial and not yet fully understood.

Due to the adverse effects described above, usage of androgens is not recommended in some certain countries. In recent years, the American Hereditary Angioedema Association Medical Advisory Committee and International Working Group of Hereditary Angioedema have specifically recommended against the use of anabolic androgens for long-term prophylaxis in pregnant or breastfeeding women, and still-developing children (generally < 16 years of age) [45, 51]. Currently, the recommended treatment option for long-term prevention of attacks in children (≥ 12 years) is C1-INH concentrate and lanadelumab. Because there are not enough safety data on the potential effects of androgens on bone maturation, growth, and development, use of attenuated androgens is not recommended in children [51]. Moreover, pregnant females should not use androgen therapy because of adverse effects, while prophylactic use of plasma-derived C1 inhibitor concentrate was found to be effective in some cases [52, 53]. Therefore, patients with HAE who are administered androgens long term should have regular physical examinations to adjust their medication regimen [1, 31]. Alternative options might be considered in certain cases, such as C1-INH concentrate for pregnant women, or C1-INH concentrate and lanadelumab for adolescents (≥ 12 years) [54].

## Quality of life (QoL) in patients with HAE

Despite advances in treatment, the burden of HAE remains heavy for patients. Indeed, there is a significant humanistic burden associated with HAE, particularly due to disease heterogeneity and the unpredictability of disabling HAE attacks, making them difficult to characterize and effectively manage [55, 56]. Attacks can occur in the absence of an identifiable event. The severity, frequency, and location of HAE attacks vary greatly both among and within patients, and are unrelated to the magnitude of C1-INH dysfunction. The intolerable experience caused by the complexity of each episode is one of the important reasons for a decline in QoL [57, 58].

The ultimate goal of HAE treatment is to ‘control’ the disease by safely preventing HAE attacks while maintaining the patient’s QoL [59]. Although treatment with danazol can slow the onset of HAE symptoms, its effects on QoL are inconsistent. According to a recent study of long-term danazol prophylaxis for HAE, no correlation was observed between QoL and danazol dose [60]. Another prospective study observed significant improvements in all QoL domains when patients were switched from long-term danazol to C1-INH for treatment [61]. This result indicates that QoL should be taken into consideration when choosing between long-term prophylactic options to achieve treatment goals.

## Safe and effective long-term prophylactic treatment alternatives

Androgens and antifibrinolytic agents are the main preventive treatment for long-term prophylaxis in Brazil and China [9, 10]. However, in consideration of the adverse effects of androgen treatment and need to improve patient QoL, androgens are no longer the first treatment choice recommended by guidelines in the global consensus [59]. The availability and cost of new treatments for HAE are important issues in China and Brazil [9, 55]. In the last decade, several new drugs and new indications for old drugs have played a role in the management of HAE. At present, other options for preventing HAE attacks primarily include C1-INH replacement therapy and kallikrein inhibitors [59, 62]. Other new drugs are also being investigated, mainly for long-term prophylaxis, and are aimed at inhibiting the kallikrein-kinin system by means of anti-prekallikrein, anti-kallikrein, and anti–activated FXII action [63].

### C1-INH

C1-INH replacement therapy [plasma-derived or recombinant human C1-INH (rhC1INH) via intravenous administration] has been recommended for short-term and long-term prophylaxis [59]. However, recent studies have shown plasma-derived C1-INH by intravenous injection every 3 or 4 days to be an effective treatment option. Following C1-INH administration, the average score of attack severity and total attack duration were significantly decreased by 32% and 38%, respectively [64]. Therefore, C1-INH provides patients with a convenient option [4].

### Lanadelumab

Lanadelumab, a human monoclonal antibody, is a plasma kallikrein inhibitor approved as a first-line therapy for long-term prophylaxis of HAE attacks. A randomized clinical trial in the United States reported that lanadelumab significantly reduced the attack rate (by 87%) in 27 patients with HAE who received 300 mg of lanadelumab every 2 weeks for at least 6 months. Twenty patients (38.1%) were completely attack-free during the stable state of treatment (days 70–182) [65]. As lanadelumab is self-administered via subcutaneous injection, it provides convenience for patients and has been associated with fewer adverse effects [66]. In the Phase 3 HELP study that included 29 subjects, improved QoL was also reported. The Angioedema Quality of Life Questionnaire (AE-QoL) includes four domain scores: functioning, fatigue/mood, fears/shame, and nutrition (Table S1). Lower AE-QoL scores reflect lower impairment and better QoL. AE-QoL scores were significantly improved in the lanadelumab group (mean change, from − 29.3 to -13.0) compared with the placebo group [67].

### Berotralstat

Berotralstat is an orally administered kallikrein inhibitor that decreases bradykinin production to prevent localized tissue edema during attacks of HAE [68]. In a double-blind, parallel-group study of 121 patients, attack rates were significantly decreased in patients administered 110 mg (1.65 attacks per month) or 150 mg (1.31 attacks per month) berotralstat compared with the control group (2.35 attacks per month) [69].

### Inhibition of FXII

Factor XII (FXII) is a key initiator of the kallikrein–kinin system, which produces bradykinin, a central mediator of angioedema, and the inhibition of FXII has become the key area in the discovery of new drugs for HAE. Recently, as a first-in-class, fully human, immunoglobulin G4 monoclonal antibody targeting activated FXII, Garadacimab (200 mg or 600 mg every 4 weeks) could significantly reduce the number of monthly attacks versus placebo and was well tolerated in a randomized, double-blind, placebo-controlled, phase 2 trial study, warranting phase 3 evaluation [70]. In brief, Table 1 demonstrated the current drugs marketed for the treatment of HAE.

**Table 1:** Drugs Marketed for the Treatment of HAE

Drug	Trademark	Mechanism	Half-life	Administration route	indications	Dose-adults	Dose-children	Regulatory status
Tranexamic acid	Amchafibrin, Cyklokapron, Lysteda	Antiplasmin & antiplasminogen activity	2-8 h	Oral, IV	LTP	1000-3000 mg/d	20-40 mg/kg/d	Europe: approved FDA: NO
Lanadelumab	Takhzyro	Kallikrein inhibition	2 weeks	SC	LTP	300 mg	Same as adults	EMA: approved FDA: approved
Danazol	Danatrol, Danocrine	Increase in hepatic C1-INH synthesis	7-12 h	Oral	LTP STP	200-400 mg/d 400-600 mg/d	2.5-40 mg/kg/d 5-20 mg/kg/d	Europe: approved FDA: approved
PdC1INH	Cinryze	C1-INH replacement	56-62 h	IV	STP On-demand treatment	1000IU Repeated 1000IU	500-1000IU Repeated 500-1000IU	EMA: approved
rhCIINH	Ruconest	C1-INH replacement	3 h	IV	On-demand treatment	≤ 84 kg: 50U/kg ＞84 kg: 4200U	Same as adults	EMA: approved FDA: approved
Icatibant acetate	Firazyr	B2R blockage	1-2 h	SC	On-demand treatment	30 mg	Adjusted by age up to 30 mg	EMA: approved FDA: approved
Ecallantide	Kalbitor	Kallikrein inhibition	1.5-2.5 h	SC	On-demand treatment	3 × 10 mg separated in 3 days	Same as adults	EMA: NO FDA: approved

**Abbreviations** IV-intravenous; SC-subcutaneous; LTP-long term prophylaxis; STP-short term prophylaxis; FDA-United States Food and Drug Administration; EMA- European Medicines Agency.

**Table S1:** AE-QoL domain structure

Domain	Items
Functioning	Impairment in any of the following: work; physical activity; spare-time activities; social relations (*4 items*)
Fatigue/mood	Difficulties in falling asleep; waking up during the night; feeling tired during the day; difficulties in concentrating; feeling downhearted (*5 items*)
Fears/shame	Feeling burdened at having swellings; fear of new, suddenly appearing swellings; fear of increased frequency of swellings; ashamed to visit public places; embarrassed by the appearance of swellings; fear of long-term negative drug effects (*6 items*)
Nutrition	General limitations in foods and eating; limitations in the selection of foods and beverages (*2 items*)

*Note* Each item has a five-point response scale ranging from 1 (never) to 5 (very often); raw scores are transformed linearly to a 0–100 scale. The questionnaire has a recall period of 4 weeks.

## Conclusion

Appropriate treatments are essential to improving the QoL of patients with HAE. Because of their wide availability and relatively low cost, androgens and antifibrinolytic agents are the main preventive treatment for long-term prophylaxis of HAE in Brazil and China. In the last decade, several options were also approved for long-term prophylaxis in emerging countries, including C1-INH (via intravenous or subcutaneous administration), lanadelumab, and berotralstat. We also summarized the mechanism of action, half-life, administration route, dosage and regulatory status of androgens, androgen-related drugs, as well as other non-androgenic prophylactic treatments used in HAE up to now. Of note, use of C1-INH is limited by the frequent dosing regimens required for intravenously administered drugs (every 3–4 days). However, subcutaneously administered pdC1-INH concentrate and lanadelumab represent significant recent advances in treatment effectiveness and improved QoL.

## Data Availability

All data generated or analysed during this study are included in this published article.

## List of abbreviations

HAE: Hereditary angioedema.
C1-INH: C1-inhibitor.
P-KK: Plasma kallikrein.
BK: Bradykinin.
FXII: Factor XII.
ANGPT-1: Angiopoietin-1.
AA: Aplastic anemia.
FPHL: Female pattern hair loss.
HDL-C: High-density lipoprotein cholesterol.
LDL-C: Low-density lipoprotein cholesterol.
QoL: Quality of life.
AE-QoL: Angioedema Quality of Life Questionnaire.
